# Treatment resistant adolescent depression with upper airway resistance syndrome treated with rapid palatal expansion: a case report

**DOI:** 10.1186/1752-1947-6-415

**Published:** 2012-12-04

**Authors:** Paul Miller, Mala Iyer, Avram R Gold

**Affiliations:** 1 807 Walt Whitman Road, Melville, NY, 11747, USA; 2 2780 Middle Country Rd, Suite 306, Lake Grove, NY, 11755, USA; 3 Stony Brook University Sleep Disorders Center, 240 Middle Country Road, Smithtown, NY, 11767, USA

## Abstract

**Introduction:**

To the best of our knowledge, this is the first report of a case of treatment-resistant depression in which the patient was evaluated for sleep disordered breathing as the cause and in which rapid palatal expansion to permanently treat the sleep disordered breathing produced a prolonged symptom-free period off medication.

**Case presentation:**

An 18-year-old Caucasian man presented to our sleep disorders center with chronic severe depression that was no longer responsive to medication but that had recently responded to electroconvulsive therapy. Ancillary, persistent symptoms included mild insomnia, moderate to severe fatigue, mild sleepiness and severe anxiety treated with medication. Our patient had no history of snoring or witnessed apnea, but polysomnography was consistent with upper airway resistance syndrome. Although our patient did not have an orthodontic indication for rapid palatal expansion, rapid palatal expansion was performed as a treatment of his upper airway resistance syndrome. Following rapid palatal expansion, our patient experienced a marked improvement of his sleep quality, anxiety, fatigue and sleepiness. His improvement has been maintained off all psychotropic medication and his depression has remained in remission for approximately two years following his electroconvulsive therapy.

**Conclusions:**

This case report introduces the possibility that unrecognized sleep disordered breathing may play a role in adolescent treatment-resistant depression. The symptoms of upper airway resistance syndrome are non-specific enough that every adolescent with depression, even those responding to medication, may have underlying sleep disordered breathing. In such patients, rapid palatal expansion, by widening the upper airway and improving airflow during sleep, may produce a prolonged improvement of symptoms and a tapering of medication. Psychiatrists treating adolescents may benefit from having another treatment option for treatment-resistant depression.

## Introduction

Treatment-resistant depression is an all-too-common occurrence among adolescents [1]. Approximately 40 percent of younger people diagnosed and being treated for depression do not respond to either serotoninergic medication or cognitive behavioral therapy. These adolescents experience impaired functioning at home and at school with increased risk of substance misuse, hospitalization and suicide, the third leading cause of death in adolescence [1]. Both the psychological and physical suffering and the lost education and socialization make treatment-resistant depression a costly disorder for the patients, their families and society.

In the following report, we present the case of a man with treatment-resistant depression whose depression and associated symptoms have been in remission for approximately two years after the diagnosis of upper airway resistance syndrome (UARS) treated with rapid palatal expansion (RPE).

## Case presentation

Our patient was an 18-year-old Caucasian man with a history of anxiety and depression managed by a psychiatrist since age 10 years. At the time his present psychiatrist took over his care, our patient, then aged 15 years, was most compromised by anxiety with frequent panic attacks and constant manifestations of anxious arousal with tachycardia, lightheadedness and sweaty palms. Our patient was also agitated and hypervigilant, feeling bullied by his schoolmates; symptoms that were exacerbated by going to school and socializing. As a result, our patient completed his last two years of high school being tutored at home. Depression, although present, was a secondary problem. Our patient was treated with serotonin reuptake inhibitors, serotonin-norepinephrine reuptake inhibitors, atypical antipsychotics, benzodiazepines and l-methylfolate, all without satisfactory control of his symptoms. At the age of 17 years, our patient’s depression worsened. He became sad and anhedonic with sleep complaints and vague threats of self-harm. His anxiety and hypervigilance continued. He was treated with lithium, fluoxetine, venlafaxine, lamotrigine and quetiapine, all without consistent improvement.

Because of the failure of medication to improve our patient’s symptoms, his psychiatrist had him evaluated by a psychiatrist specializing in electroconvulsive therapy (ECT). He received a course of ECT of three weekly treatments for eight weeks. The treating psychiatrist monitored our patient’s response and at the conclusion of ECT, our patient rated his own mood at ‘nine out of a possible 10’, 10 being the best mood. During our patient’s ECT, his parents spoke with a physician at the local sleep disorders center who suggested that, at the conclusion of his ECT, our patient be evaluated for sleep disordered breathing (SDB). One month following ECT, our patient came to our sleep disorders center for a consultation.

Our patient had mild difficulty falling asleep, taking 10 to 30 minutes. While awaiting sleep, he experienced some intense thoughts, but no restless legs. He had no trouble staying asleep. According to his parents, our patient did not snore or stop breathing, but tossed and turned throughout the night. After 10 to 12 hours of sleep, he would awaken unrefreshed with a headache and with his bed in disarray. He evaluated himself as mildly sleepy (a score of 7/24 on the Epworth sleepiness scale) with moderate/severe fatigue (a score of 5.2/7.0 on the fatigue severity scale) and somatic arousal (a score of 31/85 on the Mood and Anxiety Symptoms Questionnaire (MASQ) anxious arousal subscale, reflecting the somatic manifestations of increased sympathetic tone: for example, palpitations, sweating, tremulousness, frequent urination, feeling hot).

Our patient’s medications included lamotrigine (50mg) and quetiapine (50mg) for his anxiety, esomeprazole for gastro-esophageal reflux, cetirizine for seasonal nasal allergies and metformin for hyperglycemia.

On physical examination, our patient was found to be obese. He was 1.73m in height and weighed 101.15kg with a body mass index of 33.9kg/m2. His blood pressure of 140/85 and his pulse of 88 beats/min were both elevated. His upper airway examination revealed a narrow maxillary arch with a high arched palate, an elongated soft palate and uvula, a Mallampati score of 3 and a neck circumference of 40.64cm. The tonsils had not been removed but were not visible. The remainder of his physical examination was unrevealing.

**Figure 1:**
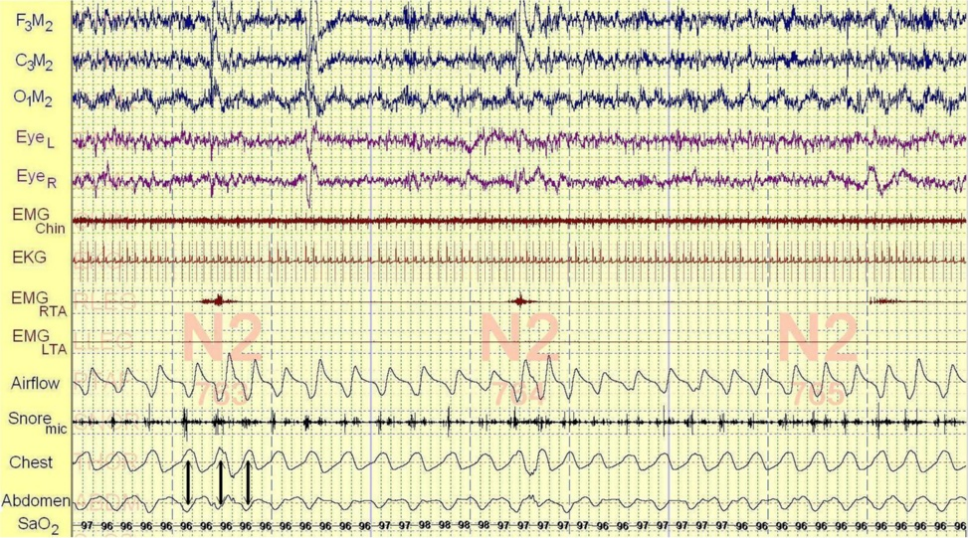
**A 90-second segment of our patient’s first polysomnogram.** This figure represents a fairly typical 90 seconds of supine NREM stage 2 sleep recorded at 4:55 a.m. of our patient’s first polysomnogram. The top three channels (F_3_M_2_, C_3_M_2_, O_1_M_2_) represent electroencephalograms. The channels Eye_L & R_ are electro-oculograms monitoring eye movements. EMG_Chin_ is a superficial electromyogram of the face muscles at the chin. Its consistently low amplitude indicates that our patient is in continuous sleep. The electrocardiogram demonstrates a heart rate of 76 beats/minute during the 90-second interval. EMG_RTA & LTA_ are superficial electromyograms of the right and left tibialis anterior muscles. The three episodes of increased activity in the EMG_RTA_ channel, in the absence of arousal from sleep, is evidence of the high frequency of periodic leg movements observed during the study. The lowest channels are monitoring respiration. The airflow channel represents inspiration as a down-going signal. The varying amplitude and duration of inspiration together with the supporting evidence of light snoring during early inspiration detected by a microphone taped to the neck (Snore_mic_) confirms the presence of mild inspiratory airflow limitation during sleep, a characteristic of both primary snoring and upper airway resistance syndrome. The belts recording respiratory effort (Chest and Abdomen) move paradoxically with respiration (three arrows) also reflecting the increased effort associated with inspiratory airflow limitation. SaO_2_ is the oxyhemoglobin saturation recorded by a pulse oximeter worn on the finger.

Polysomnography was performed eight days following his consultation. Our patient took 14.5 minutes to fall asleep and slept for 429 minutes of the 478.5 minutes he spent in bed (a sleep efficiency of 89.7 percent). His percentage of non-rapid eye movement (NREM) stage 1 sleep was increased at 15 percent of his total sleep and his REM percentage was decreased at 15 percent. During the night, our patient experienced 276 leg movements (41.8 events/hour) that were not unassociated with arousals. His apnea hypopnea index was 4.1 events/hour (below the threshold for a diagnosis of sleep apnea) and his frequency of respiratory effort-related arousals (RERAs) was 8.8 events/hour. His minimum oxyhemoglobin saturation during the night was 90 percent. Throughout the night, our patient demonstrated mild inspiratory airflow limitation intermittently associated with light snoring (Figure 1). His thoracoabdominal motion was paradoxic, consistent with his upper airway obstruction. His average heart rate during sleep was 88 beats/minute.

Based upon our patient’s history of borderline sleep-onset insomnia, restless, non-restorative sleep associated with a morning headache and moderate to severe fatigue, and his sleep study demonstrating obstructive SDB below the threshold for a diagnosis of obstructive sleep apnea, a diagnosis of UARS was made. As treatment, our patient was referred to an orthodontist for RPE, an orthodontic treatment that has been shown to relieve mild obstructive sleep apnea in both children [2] and adults [3].

**Figure 2:**
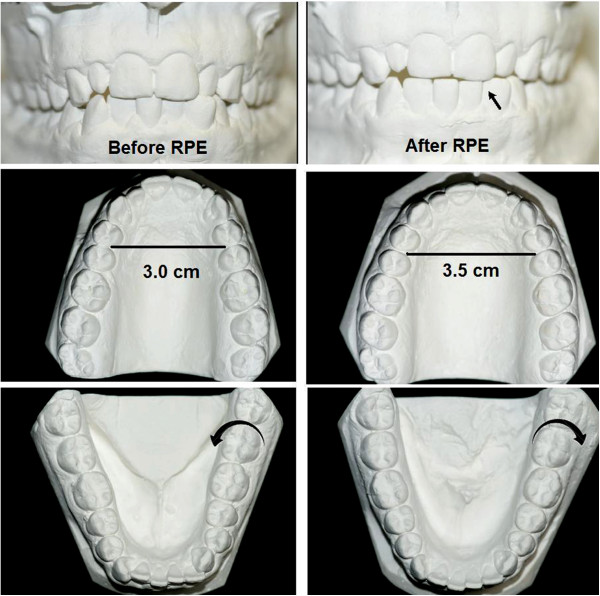
**The effect of rapid palatal expansion upon our patient’s upper airway.** This figure illustrates the effect of rapid palatal expansion on our patient’s bite. The upper panel demonstrates the opening of his bite (arrow) as the mandibular teeth tilt outwards to meet their counterparts in the expanded maxillary arch. The middle panel demonstrates the widening of the maxillary arch by 0.5cm at the level of the bicuspids after rapid palatal expansion. Notice the narrow, high palatal arch before rapid palatal expansion. The lower panel illustrates the outward tilting of the mandibular teeth following rapid palatal expansion (inward arrow before and outward arrow after rapid palatal expansion).

Upon examination, our patient’s bite was classified as Angle's Class I (he did not have a cross-bite requiring RPE for orthodontic purposes). Regardless, to treat his SDB, RPE was performed using a Haas acrylic-bearing RPE appliance. Palatal expansion continued for a period of seven weeks during which the appliance widened 7.5mm. To maintain a proper bite, a heavy labial arch lip bumper was applied to the mandibular teeth. This moved the lip away from the teeth and allowed the tongue to push the mandibular teeth outward to correct the bite (Figure 2). Two-and-a-half weeks following completion of the palatal expansion, our patient reported improved nasal breathing during wakefulness.

**Figure 3:**
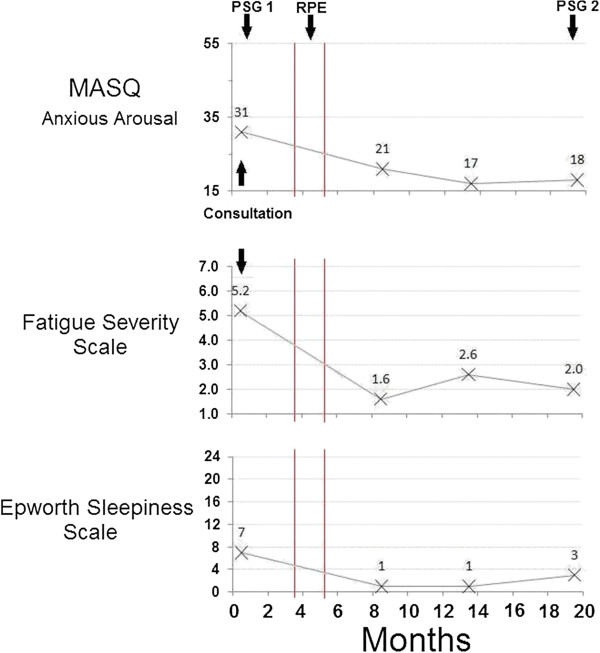
**The effect of rapid palatal expansion upon our patient’s symptoms.** This figure demonstrates our patient’s self-report assessments of somatic arousal (Mood and Anxiety Symptom Questionnaire anxious arousal subscale; MASQ Anxious Arousal), fatigue (Fatigue Severity Scale) and sleepiness (Epworth Sleepiness Scale) over time beginning at time 0, the first day of the month in which our patient presented to our sleep disorders center for consultation. The seven-week period during which rapid palatal expansion was performed is represented by vertical lines. Arrows also mark the dates of our patient’s two polysomnograms (PSG 1 and PSG 2). Somatic arousal and sleepiness decreased to normal levels during the follow-up period while fatigue decreased to the borderline between normal and mild (minimal).

Approximately four months after RPE (with the appliance still in place), our patient returned to the sleep disorders center for follow-up. He remained without symptoms of depression off antidepressant medication. His anxiety had remitted and he was no longer taking lamotrigine or quetiapine. His sleep was much improved and he was no longer awakening with a headache or complaining of fatigue. The subsequent evolution of our patient’s questionnaire scores for the Epworth sleepiness scale, fatigue severity scale and MASQ anxious arousal subscale is demonstrated in Figure 3 which illustrates the persistent improvement in our patient’s sleepiness, fatigue and somatic manifestations of increased sympathetic tone. In addition to the improvement in symptoms, our patient grew 1.27cm while losing 4.5kg in weight. His dose of metformin was halved without loss of glycemic control.

**Figure 4:**
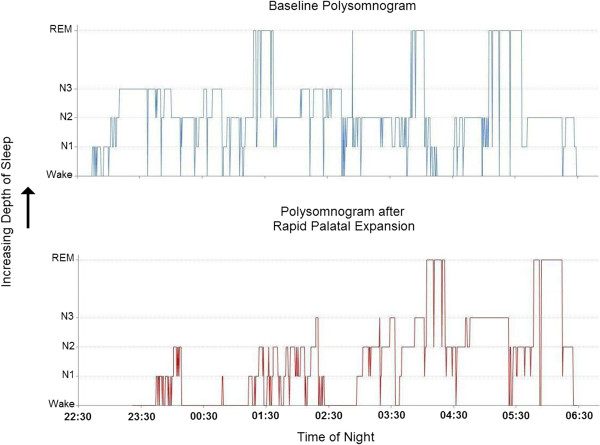
**Hypnograms before and after rapid palatal expansion.** This figure presents the sleep stage architecture (sleep stage versus time: a hypnogram) of our patient’s sleep before and after rapid palatal expansion. Sleep stages increase in depth along the ordinate from Wake to non-rapid eye movement stages 1, 2 and 3 (N1, N2 and N3) to rapid eye movement. The time of night is represented on the abscissa. During the baseline polysomnogram, our patient fell asleep quickly and slept relatively continuously throughout the night with frequent shifts between deeper and lighter sleep/wake (sleep stage shifts; a total of 70 during the night). Frequent sleep stage shifts are thought to be an adaptation to sleeping under stress (such as the presence of danger or unfamiliar surroundings; see [4]). After rapid palatal expansion, our patient had difficulty falling asleep in the unfamiliar surroundings and slept fitfully for half the night with frequent sleep stage shifts (32 during the first half of the polysomnogram). This occurrence may reflect decreased sleepiness together with the stress of his unfamiliar surroundings (at baseline, he may have been too sleepy and too stressed for the unfamiliar surroundings to matter). Beginning at approximately 3:00 a.m., our patient became acclimated to his surroundings and went into deep sleep with few sleep stage shifts compared to his baseline study at the same time of night (38 during the baseline study compared to 19 after rapid palatal expansion). The finding of decreased sleep stage shifts during the second half of the second polysomnogram may be one marker for decreased vigilance during sleep reflecting decreased stress, after rapid palatal expansion.

**Figure 5:**
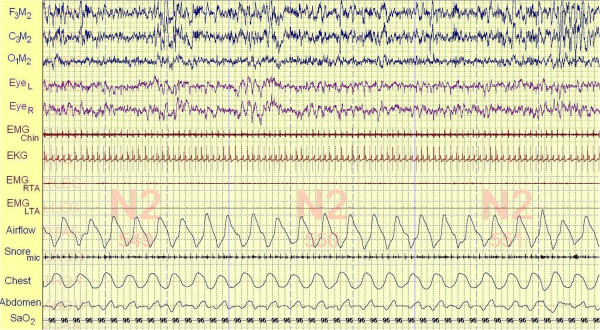
**A 90-second segment of our patient’s second polysomnogram.** This figure represents a fairly typical 90 seconds of supine NREM stage 2 sleep recorded at 4:00 a.m. of our patient’s second polysomnogram. All the channels are identical to those of the first polysomnogram (refer to the legend of Figure 1). Inspiratory airflow limitation persists in the second polysomnogram evidenced by broad, flattened inspiratory airflow signals throughout the figure (inspiration is down-going), but without audible snoring recorded by microphone (Snore_mic_; There is one audible snore at the far right of the channel). It is not possible to compare airflow values between studies because the signal cannot be precisely calibrated (the signal depends both upon sensitivity of the amplifier and upon the precise position of the pressure catheter sensing air pressure below our patient’s nose). However, based upon the principals of flow through biological tubes, decreasing nasal resistance by palatal expansion will increase maximal airflow under conditions of inspiratory airflow limitation. Furthermore, the paradoxical thoracoabdominal motion characteristic of increased inspiratory effort observed during the first polysomnogram is no longer present. Compared to Figure 1 with its heart rate of 76 beats/minute and respiratory rate of 19 breaths/minute, this figure demonstrates slowing of the heart rate to 65 beats/minute and the respiratory rate to 16 breaths/minute. The periodic leg movements that were present throughout the first polysomnogram and are evident in Figure 1 (EMG_RTA_) are absent from this figure.

**Figure 6:**
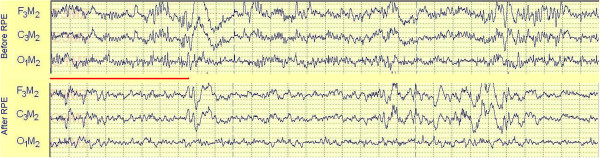
**A comparison of electroencephalographic frequencies between polysomnograms.** This figure compares two 30-second segments of NREM stage 2 sleep obtained in the early morning (3:00 a.m. to 4:00 a.m.) between our patient’s polysomnogram before rapid palatal expansion (RPE; upper panel) and after rapid palatal expansion (lower panel). Seconds are delineated by the vertical dotted lines. The three electroencephalogram channels (F_3_M_2,_ C_3_M_2,_ O_1_M_2_) in the upper panel (before rapid palatal expansion), demonstrate the presence of a 7 to 11 cycle per second oscillation (alpha frequency; a frequency associated with wakefulness) that is not prominent in the sleep of healthy individuals. In the lower panel (after rapid palatal expansion), this waking alpha frequency is decreased in amplitude (when it is seen) and the predominant frequency is three to five cycles per second (theta frequency; the frequency predominating in NREM stage 1 and NREM stage 2 sleep among healthy individuals). The horizontal red line highlights a section of the two electroencephalograms where the contrasting frequencies are well seen. The decreased presence of the alpha frequency after rapid palatal expansion suggests a decreased state of vigilance between these two polysomnogram segments.

At 16 months following RPE, with the appliance no longer in place, our patient had a post-treatment polysomnogram performed. On this occasion, he complained of being unable to fall asleep (he took 24.0 minutes) and he slept less continuously at the start of the study, suggesting that his decreased sleepiness made it more difficult for him to fall asleep in an unfamiliar bed (Figure 4). Our patient slept for 271.5 minutes of the 426.5 minutes he spent in bed (a sleep efficiency of 63.7 percent). His percentage of NREM stage 1 sleep continued to be increased at 19 percent of his total sleep and his REM percentage continued decreased at 16 percent. Figure 4 demonstrates that once our patient achieved continuous sleep (after 3:00 a.m.), his frequency of shifts from deeper to lighter sleep stages was decreased compared to his first study (his deep sleep was more consolidated). In contrast to the 41.8 leg movements/hour that were present during the first polysomnogram, no leg movements were observed during the second (Figure 5). Our patient’s apnea hypopnea index was unchanged at 4.4 events/hour and his RERA index was unchanged at 10.0 events/hour. His minimum oxyhemoglobin saturation during the night was 92 percent. The mild inspiratory airflow limitation that was present during the first polysomnogram was also present during the second (Figure 5; the use of nasal pressure as a surrogate for airflow during clinical polysomnography does not allow one to detect small changes in airflow between studies). The paradoxical thoracoabdominal motion observed during the first polysomnogram was no longer present and the inspiratory airflow limitation that was present was not accompanied by audible snoring (Figure 5). Several additional qualitative differences in our patient’s sleep were also observed between polysomnograms. Inspection of our patient’s electroencephalogram during sleep demonstrated a decrease in the waking alpha frequency (7 to 11Hz) following RPE (Figure 6). Figure 5 also demonstrates that both heart rate and respiratory rate during sleep decreased after RPE (average heart rate during sleep decreased to 76 beats/minute compared to the 88 beats/minute during the pre-treatment polysomnogram). Taken together with the decreased frequency of shifts to lighter sleep stages between studies (Figure 4), the second polysomnogram suggests a decreased level of vigilance during sleep after RPE.

Two years following his ECT, our patient remained without symptoms of depression, chronic anxiety or sleepiness/fatigue off psychotropic medication. He no longer requires home tutoring, attends college and is doing well scholastically.

## Discussion

Our patient’s case of treatment-resistant depression responding to treatment of UARS with RPE is unusual from several perspectives. First, our patient was suspected of having SDB despite having no history of snoring or witnessed apnea. Second, RPE was utilized to treat our patient’s UARS even though he had no cross-bite, the condition for which RPE was developed. Third, following RPE, our patient’s apnea hypopnea index and frequency of RERAs (indices of SDB severity) did not change, yet his anxiety, sleepiness and fatigue improved markedly and his depression remained in remission without medication. What is the evidence that his UARS improved and how did that improvement lead to a change in his symptoms? These issues will be discussed below.

To consider that unrecognized SDB may underlie depression is not standard medical practice. Yet, increasing evidence suggests that SDB and depression are associated in both children [5] and adults [6]. Furthermore, in children, the association does not appear to depend upon the severity of pharyngeal collapse during sleep. A history of pronounced snoring is enough to consider a person at increased risk for depression [5]. Therefore, our patient’s diagnosis of UARS would put him at increased risk for depression.

UARS is an association between very mild pharyngeal collapse during sleep, a severity of collapse that does not meet the clinical threshold for a diagnosis of sleep apnea, and daytime sleepiness/fatigue [7]. Sleep-onset insomnia is also frequently associated [8]. While collapse of the pharyngeal airway during sleep occurs in all patients with UARS, not all patients with UARS snore audibly [9]. Therefore, anyone with sleep-onset insomnia, restless sleep and daytime sleepiness/fatigue, symptoms that are common among depressed individuals, may have UARS.

Our treatment of our patient’s UARS with RPE is consistent with the literature demonstrating an improvement in mild sleep apnea among children treated with RPE [2]. However, RPE to treat an adolescent with SDB in the absence of a cross-bite has not been previously reported. We reasoned that our patient’s Angle’s Class I bite was not acceptable in the presence of UARS and treatment resistance depression, so widening his nasal airway while tilting the mandibular teeth outward to correct his bite was needed, and successfully achieved.

The idea that RPE improved our patient’s inspiratory airflow alleviating UARS is central to this case report. But what is the evidence that airflow improved? Inspiratory airflow limitation (IFL) persisted in our patient’s second polysomnogram and the airflow signal, which was uncalibrated, did not allow for quantitative comparisons. While the direct evidence for increased inspiratory airflow is missing, the equation for flow through the upper airway during IFL (maximal flow=-(pharyngeal critical pressure)/upstream resistance, where the upstream airway is from the nares to the point of pharyngeal collapse) implies that widening the nasal airway led to an increase in maximal inspiratory airflow through decreased nasal resistance [10]. The resolution of the paradoxical thoracoabdominal motion observed during polysomnography after RPE provides indirect evidence for improved inspiratory airflow that decreased inspiratory effort. Therefore, without direct evidence, it is reasonable to conclude that RPE improved our patient’s inspiratory airflow despite the persistence of IFL during sleep.

While an association between SDB and depression is increasingly recognized, the mechanism for that association is unclear. Although previous investigators have hypothesized contributions from fragmented sleep, hypoxemia and hypercapnia [5,6], RPE did not decrease our patient’s sleep fragmentation nor did it prevent hypoxemia, which was not present before treatment. An alternative hypothesis is that, for our patient, mild IFL during sleep was a stressor that chronically activated his brain’s limbic system, sympathetic nervous system and hypothalamic-pituitary-adrenal axis [11]. Such an hypothesis can explain the mild sleep-onset insomnia, increased vigilance during sleep, anxiety, sleepiness/fatigue and depression experienced by our patient (symptoms of chronic stress [4,11]) while explaining his growth and improved glycemic control (increased growth hormone and decreased cortisol [11]) following RPE.

A further clue to understanding the resolution of our patient’s depression and anxiety is found in a comparison of his questionnaire data and polysomnography before and after treatment. Before treatment, our patient had increased sympathetic nervous system activity manifest as an increased score on the MASQ anxious arousal subscale and an increased heart rate during sleep. After RPE, our patient’s score on the MASQ anxious arousal subscale was minimized (Figure 3) and his heart rate during sleep decreased. Furthermore, before treatment, our patient’s polysomnogram demonstrated marked periodic leg movement disorder that was gone following treatment. Periodic leg movement disorder is believed to reflect central nervous system dopamine deficiency [12]. Thus, treatment of our patient’s UARS may have altered his central catecholamine balance decreasing sympathetic nervous system mediators while increasing central dopamine. Increased central dopamine activity has been associated with a decreased tendency toward depression [13] and anxiety [14] in humans.

Because our patient’s depression was treated with ECT and remitted before he underwent RPE, we are left with some uncertainty about the contribution of RPE toward our patient’s present affective state. How do we know our patient is without symptoms because of RPE? Although there is limited information concerning the effect of ECT in adolescents, in the one case series of 10 patients treated with ECT, most were maintained on one or two antidepressants post-ECT and three of the nine followed for one year relapsed [15]. Our patient’s psychiatrist has treated one other depressed adolescent with ECT. That patient underwent a remission that was maintained with an antidepressant for one year before relapse. In the literature, there is no discussion of the effect of ECT on the sleep complaints or anxiety that may be associated with major depression. In our patient’s case, both were present after ECT and resoloved after RPE.

## Conclusions

The sustained improvement of this adolescent’s treatment-resistant depression, anxiety and fatigue/sleepiness after RPE is a clinical finding of enormous importance to psychiatrists managing depression and their patients. The symptoms of SDB can be so non-specific (fatigue, insomnia, restless sleep) that every depressed adolescent may have them. Indeed, the prevalence of IFL during sleep among depressed adolescents is unknown. However, given the physically benign nature of RPE as a treatment and the control that may be obtained over symptoms, we believe that evidence of SDB should be sought in every depressed adolescent and, when present, treated and the resulted should be documented.

## Consent

Written informed consent was obtained from the patient for publication of this case report and any accompanying images. A copy of the written consent is available for review by the Editor-in-Chief of this journal.

## Competing interests

The authors declare that they have no competing interests.

## Authors’ contributions

PM performed our patient’s rapid palatal expansion. MI provided our patient’s psychiatric care. ARG performed the sleep consultation, interpreted the two polysomnograms and provided the follow-up care for our patient’s upper airway resistance syndrome. All authors read and approved the final manuscript.
